# Significant Subgraph Detection in Multi-omics Networks for Disease Pathway Identification

**DOI:** 10.3389/fdata.2022.894632

**Published:** 2022-06-22

**Authors:** Mohamed Abdel-Hafiz, Mesbah Najafi, Shahab Helmi, Katherine A. Pratte, Yonghua Zhuang, Weixuan Liu, Katerina J. Kechris, Russell P. Bowler, Leslie Lange, Farnoush Banaei-Kashani

**Affiliations:** ^1^Big Data Management and Mining Laboratory, Department of Computer Science and Engineering, College of Engineering, Design and Computing, University of Colorado Denver, Denver, CO, United States; ^2^Department of Mathematics, College of Liberal Arts and Sciences, University of Colorado Denver, Denver, CO, United States; ^3^National Jewish Health, Denver, CO, United States; ^4^Department of Biostatistics and Informatics, Colorado School of Public Health, University of Colorado Anschutz Medical Campus, Aurora, CO, United States; ^5^School of Medicine, University of Colorado Anschutz Medical Campus, Aurora, CO, United States; ^6^Division of Biomedical Informatics and Personalized Medicine, Department of Medicine, University of Colorado Anschutz Medical Campus, Aurora, CO, United States

**Keywords:** graph clustering, PageRank, Louvain, multi-omics graph, subgraph detection

## Abstract

Chronic obstructive pulmonary disease (COPD) is one of the leading causes of death in the United States. COPD represents one of many areas of research where identifying complex pathways and networks of interacting biomarkers is an important avenue toward studying disease progression and potentially discovering cures. Recently, sparse multiple canonical correlation network analysis (SmCCNet) was developed to identify complex relationships between omics associated with a disease phenotype, such as lung function. SmCCNet uses two sets of omics datasets and an associated output phenotypes to generate a multi-omics graph, which can then be used to explore relationships between omics in the context of a disease. Detecting *significant subgraphs* within this multi-omics network, i.e., subgraphs which exhibit high correlation to a disease phenotype and high inter-connectivity, can help clinicians identify complex biological relationships involved in disease progression. The current approach to identifying significant subgraphs relies on hierarchical clustering, which can be used to inform clinicians about important pathways involved in the disease or phenotype of interest. The reliance on a hierarchical clustering approach can hinder subgraph quality by biasing toward finding more compact subgraphs and removing larger significant subgraphs. This study aims to introduce new significant subgraph detection techniques. In particular, we introduce two subgraph detection methods, dubbed Correlated PageRank and Correlated Louvain, by extending the Personalized PageRank Clustering and Louvain algorithms, as well as a hybrid approach combining the two proposed methods, and compare them to the hierarchical method currently in use. The proposed methods show significant improvement in the quality of the subgraphs produced when compared to the current state of the art.

## Introduction

Discovering significant subgraphs within multi-omics networks, such as those produced by methods such as sparse multiple canonical correlation network analysis (SmCCNet) (Shi et al., 2019) and DIABLO (Singh et al., 2019), can be a valuable tool for gaining information about disease pathways. Such subgraphs can inform clinicians about molecules which interact with each other and play a role in the progression of a disease of interest. An ideal subgraph is highly correlated to a disease phenotype and contains nodes which are highly related to each other's function or expression. Currently, subgraphs are identified using hierarchical clustering, which comes with some limitations, such as a bias toward small, uniform subgraphs, and poor scalability to larger biological networks. We propose three new methods for detecting significant subgraphs, which improve the quality of subgraphs produced while being more easily scalable to large datasets. In the rest of this section, we introduce a motivating application of the methods we introduce, then provide an overview of the current approach to significant subgraph detection, and conclude with an overview of our contributions.

### Motivating Application

Chronic obstructive pulmonary disease (COPD) is a chronic lung condition which causes expiratory airflow limitation and respiratory problems, and has recently been the fourth leading cause of death in the United States (Garcia et al., 2017). There are a variety of environmental, behavioral, and genetic factors that contribute to development of this disease. Clinical variables, such as age, smoking history, body mass index (BMI), and dyspnea, are often used for modeling and predicting disease severity and progression. Nevertheless, due to the highly complex and widely heterogeneous phenotypes of COPD, the complex relationship between these variables is not well understood (Reinhold et al., 2017; Zemans et al., 2017).

COPD is independently associated with systemic diseases such as coronary artery disease, congestive heart failure, interstitial lung disease, pulmonary hypertension, cancer, weight loss, metabolic syndrome, and diabetes and blood is commonly used to study the systemic effects of COPD. Blood biomarkers are often used to assess the relationship between smoking and COPD. Most biomarker studies have focused on single molecules, as they can facilitate prognosis and individualized treatment. However, as single biomarkers cannot fully explain the COPD cross-sectional and longitudinal outcomes, recent studies suggest multiple biomarkers may be more informative to predict severity, progression, and morality (Zemans et al., 2017; Mastej et al., 2020).

We will use COPD as a running example to demonstrate our proposed methods. We introduce three new methods for the detection of significant subgraphs in a protein-metabolite multi-omics network, which we compare to the current state of the art approach.

### Current State of the Art

SmCCNet uses canonical correlation analysis (CCA) to calculate correlation between a pair of biomarkers or omics features, such as proteins and metabolites, in the context of a disease marker, such as forced expiratory volume in 1 s (FEV_1_) or forced expiratory volume in 1 s percent predicted (FEV_1_%) (Mastej et al., 2020). SmCCNet produces a graph representing the interaction of the chosen biomarkers and their correlation with the disease marker. Combined with network analysis tools, these graphs become a powerful way to explore the interaction of multiple biomarkers and a target disease. Network clustering techniques can be used to detect communities relevant to disease progression within a graph. Groups of relevant biomarkers can be used to inform further research into disease progression or targeted therapies.

Currently, hierarchical clustering is commonly used to identify omics clusters or modules in methods such as Weighted Gene Co-Expression Analysis (WGCNA) (Langfelder and Horvath, 2008). Hierarchical clustering is a limited approach to clustering graphs produced by SmCCNet, as it does not consider correlation to the phenotype, clustering based solely on the topology of the network. While edges between nodes are weighted according to their correlation together and to the phenotype, these weights only reflect that relationship between a pair of connected nodes and the phenotype. Edge weights alone fail to reflect the broader relationship between a larger group of nodes and the target phenotype, and thus a clustering algorithm which only considers topology is inadequate. Furthermore, hierarchical clustering, and most other clustering methods, aim to assign every node in a graph to a cluster, whereas significant subgraph detection simply wants to find an important subset of nodes within the graph. Finally, this approach does not scale to larger omics networks, and can quickly become computationally expensive when applied to typical biological dataset.

### Contribution

In this paper, we present three approaches to detecting significant subgraphs in networks produced by SmCCNet.

We first introduce an extension of the Personalized PageRank Clustering (PPC) algorithm, dubbed Correlated PageRank, to find significant subgraphs. PageRank is a local graph clustering method that is commonly used for web page ranking, social networks, and recommendation systems (Tabrizi et al., 2013; Xie et al., 2015). This method offers better scalability when compared to hierarchical clustering, as its runtime is determined by the size of the identified subgraph rather than the global graph. We extend PPC by incorporating correlation information into the subgraph identification process, introduced in more detail in Section Correlated PageRank.

We then introduce an extension to the Louvain algorithm, similarly incorporating correlation to identify significant subgraphs. The Louvain algorithm offers a scalable approach to identifying subgraphs in a network by applying repeated objective optimization and merging steps, and has been shown to offer good scalability while maintaining subgraph quality. Our contribution, dubbed Correlated Louvain, is introduced in more detail in Section Correlated Louvain.

Finally, the two proposed methods were combined into a hybrid approach, utilizing Correlated Louvain to produce high quality seed nodes to form the teleport set for the Correlated PageRank algorithm, and iteratively applying the two methods to produce highly correlated, succinct subgraphs. We detail this approach in Section Hybrid Approach.

In addition to the subgraph identification techniques, we also developed a novel technique to visually compare subgraphs produced by these methods. This subgraph comparison technique allows for the highlighting of *unique* subgraphs from a pool of identified significant subgraphs, reducing overlap between subgraphs and producing a more informative set of subgraphs.

To compare performance of the proposed measures, the top subgraphs produced by the proposed methods are compared to those produced by the currently used hierarchical clustering approach. We show that our proposed approaches to subgraph identification outperform the currently accepted state of the art, producing more highly correlated subgraphs.

The remainder of the paper is organized as follows: Section Related Work examines related work. Section Problem Definition formalizes the problem of subgraph identification. Section Methods describes the overall pipeline as well as a more in-depth description of the three approaches. Section Experimental Evaluation: A Case Study evaluates the performance of the proposed approaches in the form of a case study. Finally, Section Conclusion and Future Work offers concluding remarks and future directions for this work.

## Related Work

In what follows, we briefly review the field of graph clustering, then give an overview of SmCCNet, a tool which has been used to generate multi-omics networks, which present one potential application of subgraph detection.

### Graph Clustering

Graph clustering is the process of finding subgraphs of related nodes within a graph (Schaeffer, 2007). This field has grown recently, and has seen many applications, from community detection in social networks, to ranking search results, to powering recommendation systems. The field of graph clustering algorithms is vast, but methods revolve around the optimization of an objective function which aims to quantify the connectivity within a subgraph compared to the connectivity to nodes outside of the subgraph. That is, graph clustering algorithms aim to find subgraphs which maximize connectivity within the subgraph, while minimizing connectivity to nodes outside the subgraph. Graph clustering methods can generally be split into two broad categories, global and local (Schaeffer, 2007).

Global clustering methods aim to assign every node in a graph to a cluster. Global clustering can either produce a hierarchical structure, where a hierarchy of clusters is generated with each top-level cluster being composed of subclusters, each of which can be its own top-level cluster to a set of subclusters, or it can produce a flat clustering where each cluster is simply a vertex subset of the global graph. Hierarchical clustering methods can be further classified as divisive or agglomerative.

Divisive global methods, or top-down methods, recursively partition a graph into clusters, starting with the global graph. Different criteria have been developed to determine how a graph should be divided, such as min-cut methods which find the minimum cut within the graph to maximize flow (Elias et al., 1956), or similarity based metrics that split the graph in a way which optimizes some similarity metric such as conductance or modularity. Spectral methods which use the eigenvectors of the normalized Laplacian, or approximations thereof, as a similarity measure have also been proposed (Capocci et al., 2005; Qiu and Hancock, 2006). Random walk approaches find clusters on the principle that a random walker is likely to stay within a cluster than it is to hop between clusters (Chen et al., 2008; Zhang et al., 2016). Meila et al. have in turn shown a relationship linking the mathematics of random walks to those of cut-based methods (Meil and Shi, 2000).

Agglomerative global methods, also known as bottom-up approaches, operate on the opposite end of the spectrum. Instead of dividing graphs into subgraphs, they merge similar subgraphs together to form larger graphs. Similarity measures such as Jaccard index and cosine similarity, or connectivity measures such as modularity are used to determine suitable subgraphs to merge. Carrasco et al. propose one such method to bipartite graphs (Carrasco et al., 2003). The Louvain algorithm is a popular global agglomerative graph clustering algorithm (Blondel et al., 2008). The Louvain algorithm is an agglomerative clustering strategy that iteratively moves nodes to neighboring clusters to locally maximize modularity, then merges the cluster to create a representative super-node to be considered in the following iteration. It aims to improve scalability without significantly reducing partitioning quality. It has been used in a variety of applications, such as clustering twitter users based on their political preferences (Sánchez et al., 2016), community detection based image segmentation (Nguyen et al., 2018), and recently used to classify aquatic biota (Milošević et al., 2022). Owing to the use of modularity during the optimization step, the Louvain algorithm is subject to the resolution limit, where communities smaller than some threshold can't be detected (Fortunato and Barthélemy, 2007). Louvain is also limited due to the algorithms inability to move nodes once they have been merged into a super-node in a previous iteration, which can lead to non-optimal segmentations with certain network topologies (Traag et al., 2019). This algorithm has been expanded upon over time, making it more parallelizable (Lu et al., 2015) and introducing additional heuristics to improve runtime and address some of the limitations of the original algorithm (Traag et al., 2019). Despite these limitations, the base algorithm remains a widely used method due to its simplicity and applicability to a large variety of problems.

Traditionally, global clustering techniques generate hard partitionings where each node belongs to one cluster. However, this restriction isn't representative of some applications. For example, nodes in a biological graph may represent molecules with multiple functions, which wouldn't be highlighted with a traditional clustering approach. Overlapping clustering techniques allow nodes to belong to multiple clusters (Baadel et al., 2016). Several variations of K-Means clustering have been proposed. Fuzzy K-Means uses membership degrees to assign membership to different clusters, allowing a data point to belon to multiple clusters simultaneously, with different weights (Peizhuang, 1983). Overlapping K-Means introduces a threshold to determine whether an object assigned to a cluster should also be assigned to its next nearest neighbor (Cleuziou, 2009). Weighted Overlapping K-Means extends Overlapping K-Means and Weighted K-Means which introduces a weighting vector when considering distance and importance of certain features (Huang et al., 2005). Overlapping clustering approaches have also been applied to graph clustering. LP-HCLUS is one such method, generating hierarchical clusters with potential for overlap, allowing diseases and ncRNA in a heterogenous graph to be involved in multiple interaction subnetworks, better reflecting their true function (Barracchia et al., 2020).

Local clustering methods aim to find important clusters within a larger subgraph, rather than assigning every node to a cluster. This is important for larger graphs, as global methods tend to be computationally expensive or unfeasible. Local search methods are designed to find near-optimal solutions without exploring the entirety of the graph. One such method, Personalized PageRank Clustering (PPC), has previously been used for complex disease analysis. PPC calculates PageRank scores for each node in a graph, then uses those scores to identify a community around a user-specified seed node. Kohler et al. used it to search in the interactome for the prioritization of candidate disease genes (Kohler et al., 2008). Voevodski et al. applied PageRank-Nibble to identify local communities in protein-protein interaction networks (Voevodski et al., 2009). They demonstrated that PPC outperforms other partitioning methods such as spectral and nearest neighbor, finding clusters which are more coherent and better connected, measured by conductance, while also being biologically relevant. Shang and Liu leveraged weighted PPC on bilayer molecular networks to prioritize Type 2 diabetes genes (Shang and Liu, 2021). Li and Zhao utilized a multiplex PPC approach to mine functional modules in gene-gene and protein-protein networks (Li and Zhao, 2016).

Biclustering approaches add another dimension to the clustering problem. While clustering algorithms assign samples into clusters, biclustering approaches simultaneously assign samples and their features into clusters, offering more flexibility (Busygin et al., 2008). Given a matrix of genes and conditions, for example, clustering approaches would either produce gene clusters or condition clusters, whereas biclustering approaches can cluster both together, producing arbitrary subsets of genes and conditions. Direct clustering, the first of these methods, relies on statistics analysis of submatrices to form biclusters (Hartigan, 1972). Cheng and Church's node-deletion algorithm introduced the mean squared residue measure and heuristics to minimize it and obtain a gene expression bicluster (Cheng and Church, 2000). BROCCOLI uses matrix factorization to produce biclusters which allow for overlapping clusters, as well as being robust against outlier data points (Hess et al., 2021).

One primary limitation with these general clustering techniques is that they fail to incorporate external data, solely clustering based on topology. While topology is important, other factors, such as correlation of the nodes to a target variable, have no effect on clustering outcomes. Attributed network clustering techniques have previously been proposed to address this shortcoming (Chunaev et al., 2020). Early fusion methods, such as that proposed by Bhatt et al., merge node attributes and graph structure together before applying a clustering algorithm (Bhatt et al., 2019). Simultaneous fusion methods combine the clustering and fusion steps such that attribute and structure information are combined during the community detection process. One such method, PLANE, uses a generative process to model a document's embedding and topic simultaneously, then uses a mapping function to correlate documents and topics. Late fusion methods apply community detection separately to structure and attribute data, then combine the partitions to generate a structure-and-attribute aware partitioning. While these techniques address the problem of integrating structure and attribute data into an inclusive partitioning, they do not consider correlation between node attributes and an external target. Clusters identified using these methods will be similar structurally and attribute-wise, but they may not be relevant to the application as that relevance was not taken into consideration.

Network based approaches have been used to characterize biomarker-disease interactions in the past. Yang et al. applied a propagation algorithm to a bipartite lncRNA-disease graph to discover hidden lncRNA-disease associations (Yang et al., 2014). Similarly, Alaimo et al. applied a resource transfer technique to a tripartite graph to predict nrRNA-target and target-disease interactions (Alaimo et al., 2014). Both of these methods exploited known interactions to produce bipartite and tripartite graphs, respectively, which were then used to predict candidate interactions that could be tested *in vitro*. While these are powerful techniques for identifying single biomarkers which may be associated with a disease, they don't capture the interaction between larger groups of biomarkers in a disease pathway, and they don't represent multi-omic interactions.

The problem of significant subgraph detection necessitates incorporating not only structural and attribute information within the graph but doing so in the context of an external target variable. In the context of COPD networks generated by SmCCNet, a significant subgraph is one whose nodes are similar structurally and attribute-wise while also being highly correlated to a selected phenotype such that they are clinically relevant. Existing methods fail to represent this relationship to a target variable. To address this, we introduce two methods based on a local clustering approach and an agglomerative global approach, namely Personalized PageRank Clustering and Louvain, respectively, which we extend beyond the base algorithms to incorporate correlation to a target variable. We also introduce a hybrid method combining the two approaches to obtain higher quality subgraphs.

### Significant Subgraph Identification

SmCCNet is a tool used for integrating multiple omics datasets together with a phenotype of interest to generate a multi-omics graph representing the interaction between biological markers in the context of the phenotype. It uses canonical correlation analysis to integrate two sets of omics data and a quantitative disease phenotype, producing a graph with nodes representing different biomarkers and edges between them representing canonical correlation between the connected pair of biomarkers in the context of the phenotype. These graphs can be used for downstream tasks such as community detection, which can help inform clinicians about pathways important to a disease's progression. SmCCNet has previously been used by several authors for omics network detection. Zhuang et al. applied the method on miRNA-mRNA networks to identify the relevant omics features for COPD phenotypes in a set of 404 subjects (Zhuang et al., 2021). Mastej et al. used SmCCNet to explore novel protein and metabolite networks related to lung function and emphysema (Mastej et al., 2020).

Currently, detecting important subgraphs from a multi-omics graph is done using hierarchical clustering. While this approach does lead to the detection of well correlated graphs, it suffers from two major limitations. Hierarchical clustering can bias toward smaller, similarly sized clusters, ignoring larger but potentially more important pathways. Hierarchical clustering also doesn't scale well to larger omics networks, which is particularly limiting with typical biological datasets.

## Problem Definition

In this section, we give a formal definition of the problem. Let *G* = (*V, E*) be a weighted undirected graph with vertex set *V* and edge set *E*. We assume that *G* is a simple graph, with no multiple edges connecting nodes. We define edge set *E* = {((*v*_*i*_, *v*_*j*_), *w*) | *v*_*i*_, *v*_*j*_, ∈*V*} , where *w* = *f*(*v*_*i*_, *v*_*j*_), 0 ≤ *w* ≤ 1 represents an edge weight defined by a function *f*. Each node *v*_*i*_ ∈ *V* has a corresponding data vector $x_{i}\in R^{m}$. For a set of *n* vertices *V*, we define a data matrix $X=\left[x_{1}^{T},\;x_{2}^{T},\;\ldots ,\;x_{n}^{T}\right]$, the concatenation of the transpose of the data vector corresponding to each node in *V*. We also define a target vector, *Y* ∈ *R*^*m*^.

Let *G*′ = (*S, E*′) be a subgraph of *G*, where *S* ⊆ *V* and $E^{{\;}^{\prime }}=\left\{\left(\left(v_{i},\;v_{j}\right),\;w\right)\;|\;v_{i},\;v_{j},\;\in S\right\},\;E^{{\;}^{\prime }}\subseteq E$, with data matrix *X*′. The correlation ρ of a subgraph is defined as the Pearson correlation between the first principal component of the data matrix *X*′ corresponding to the set of nodes *S*, *PC*1_*s*_, with the target variable *Y*, as shown below in Equation 1. *PC*1_*s*_ is used to represent the data matrix *X*′ as a single vector to facilitate comparison to the target variable. While there are other measures that can be used to represent correlation with a target variable, such as Spearman correlation or Kenall correlation, we chose Pearson correlation to match the measure used by Mastej et al. to facilitate comparison between findings (Mastej et al., 2020). The cohesion of subgraph *G*′ can be calculated using various connectivity measures, such as modularity or conductance, and serves as a measure the connectivity of subgraph *G*′ relative to *G*. These measures are specified further in Section Methods.


(1) $$\begin{array}{l} \rho \;\left(PC1_{S},\;Y\right)=\frac{\sum_{i\in PC1_{S},\;j\in Y}\left(i-\bar{PC1_{S}}\right)\left(j-\bar{Y}\right)}{\sqrt{\sum_{i\in PC1_{S},\;j\in Y}{\left(i-\bar{PC1_{S}}\right)}^{2}{\left(j-\bar{Y}\right)}^{2}}} \end{array}$$

We define a significant subgraph as the subgraph *G*′ = (*S, E*′) with maximal correlation and cohesion among all subgraphs of *G*. With the *significant subgraph detection* problem, given a graph *G*, we want to find a significant subgraph which optimizes a bi-criteria function reflecting correlation and cohesion, such that a significant subgraph is maximally correlated to *Y* while also maximally cohesive topologically. A subgraph identified by significant subgraph detection, relative to other subgraphs, will have high connectivity within the subgraph, low connectivity outside of the subgraph, and high correlation to the target variable *Y*.

As defined above, significant subgraph detection is a constrained graph clustering problem with correlation as the constraining balancing factor, and considering any balancing factor for graph clustering has been shown to be an NP-Hard optimization problem (Wagner and Wagner, 1993). In this paper, we introduce efficient heuristic methods as approximate solutions for significant subgraph detection problem.

## Methods

We will first summarize the basic PPC and Louvain algorithms below, then describe the changes we made to incorporate correlation to the phenotype to formally define Correlated PageRank and Correlated Louvain. We also discuss a hybrid approach which combines Correlated PageRank and Correlated Louvain to produce more refined subgraphs. We then present our approach to comparing subgraphs produced by the proposed approaches. We conclude by briefly discussing the platform used to implement and test these methods.

### Correlated PageRank

We begin by giving an overview of the existing PPC algorithm. PPC is a local graph clustering method that is used in a variety of applications, such as webpage ranking, social networks, and recommendation systems (Tabrizi et al., 2013; Xie et al., 2015). It offers better scalability with runtimes proportional to the size of individual clusters rather than the full graph. This algorithm works based on the Markov process; a random walker starts at a seed node and travels through nodes in the graph either by transitioning to a neighboring node, with probability 1−α, or by teleporting to a node in the defined teleportation set independent of its current location, with probability α. The PageRank formula is shown in Equation 2 (Gleich and Kloster, 2016); *pr*_α_(*S*) is the steady state distribution of a random walker, α ∈ (0, 1) specifies the teleportation probability, s is the teleportation set, and W is the random walk transition matrix (Voevodski et al., 2009).


(2) $$\begin{array}{l} pr_{\alpha }\;\left(s\right)=\alpha s+\left(1-\alpha \right)\;pr_{\alpha }\;\left(s\right)\;W \end{array}$$

The steady state vector, which is also called the PageRank vector, is used to sort the vertices from higher to lower probability-per-degree, which represents the probability of a node being visited by the random walker. This produces the sweep-set, a set of *m* non-zero entries in the vector set *V*. The sweep cut procedure computes the conductance of the first *j* elements (from 1 to *m*) in the sweep-set, and the set with lowest conductance is selected.

PPC results are affected by a variety of user-selected parameters. The seed set determines the nodes around which a cluster is formed. Selection of the set of seed nodes can be done based on node degree, with high degree nodes being selected to act as hub nodes, or the selection can be informed by biological function, with nodes corresponding to biological markers suspected of being highly correlated to the disease being selected. The teleportation probability affects the distance from the seed set the random walker will explore, with a higher teleportation probability resulting in a higher emphasis on near the seed set, and a lower probability producing larger diameter clusters. The termination factor (ϵ ∈ [0, 1]) determines the length of the search domain.

The traditional PPC approach to clustering lacks consideration for correlation to the phenotype. While subgraphs identified by the algorithm will be densely connected, this does not capture the full relationship between omics and the phenotype. In order to better identify important subgraphs, correlation between a given set of omics and the phenotype is calculated and incorporated. Correlated PageRank consists of two main steps, a subgraph identification step where significant subgraphs are identified based on the calculated PageRank score, and a pruning step which serves to decrease the size of subgraphs produced such that the algorithm produces smaller subgraphs while maintaining connectivity and correlation. In Section Subgraph Identification we introduce two approaches to incorporating correlation into the subgraph identification step of Correlated PageRank, dubbed *sequential* and *simultaneous*. In Section Subgraph Pruning we introduce two approaches to pruning the subgraphs produced in the previous step, which we refer to as *threshold* and *nesting*. The nesting approach to pruning is further split into two approaches, called *graph nesting* and *subgraph nesting*.

#### Subgraph Identification

Two measures were used when identifying significant subgraphs, conductance (Φ) and omics-phenotype correlation (ρ). We define volume of *G* to be the sum of degrees of the nodes in *G*, shown in Equation 3. Conductance, shown in Equation 4 (Gleich and Kloster, 2016) below, is a measure of how well connected a subgraph with nodes *S* is relative to the global network with nodes *V* and edge weights *w*. A lower conductance represents a better subgraph that is well connected internally and well isolated from the remainder of the graph.


(3) $$\begin{array}{l} vol\;\left(G\right)=\underset{j\in G}{\sum }d_{j} \end{array}$$

(4) $$\begin{array}{l} \text{Φ}\;\left(S,\;V,\;w\right)=\;\frac{\sum_{i\in S\;,\;j\notin S}wij\;}{\min\left(vol\left(S\right),\;vol\left(V-S\right)\right)} \end{array}$$

Two methods were used for subgraph identification, referred to as sequential and simultaneous. In the sequential approach, the conductance (Φ) was calculated during the sweep cut procedure to generate highly connected subgraphs. The omics-phenotype correlation (ρ) was then calculated for each subgraph as in Equation 1, and those with the highest correlation were chosen as the best subgraphs. In the simultaneous approach, conductance and correlation are combined into a single objective function as a weighted sum, shown in Equation 5, replacing conductance during the sweep cut procedure. *k*_*P*_ is a user set weight, such that *k*_*P*_ ≤ 1, which sets the proportion of conductance and correlation in the combined objectective function Φ+ ρ.


(5) $$\begin{array}{l} \text{Φ}+\rho =k_{P}*\text{Φ}+\left(1-k_{P}\right)*\rho \; \end{array}$$

#### Subgraph Pruning

Subgraphs produced by Correlated PageRank are very large and may contain weak edges between features. To improve subgraph quality, we explored methods for shrinking the size of the identified subgraph, removing weaker edges and improving correlation. Two pruning methods are applied, threshold and nesting.

In the threshold pruning approach, a threshold is chosen, and any edges with an edge weight lower than the threshold are removed. Isolated nodes and smaller fragments resulting from this edge removal are also removed from the subgraph, resulting in a smaller, more strongly correlated subgraph. While a similar pruning approach is applied during the SmCCNet procedure to produce the global graph, the thresholds used are intentionally small to avoid over-pruning and fragmenting the network prior to downstream analyses. To choose the optimal threshold, a grid search approach is used. Different thresholds in the range of edge weights are tested, with the optimum threshold resulting in the highest omics-phenotype correlation and an appropriate ratio between omics, informed by the biological context of the omics and disease of interest. This approach to pruning is naïve, as it removes all edges classified as weak without considering their effects on the overall quality of the subgraph. While a grid search technique was applied to find an optimal threshold that led to the highest quality subgraph, this remains a heavy-handed approach to decreasing subgraph size.

In the nesting approach, the Correlated PageRank algorithm is iteratively applied to produce progressively smaller subgraphs. Two nesting sub-approaches were investigated, graph nesting and subgraph nesting. In graph nesting, once a top subgraph *G*′ was identified, it is removed from the global network *G*, and Correlated PageRank was applied to the resulting network *G*\*G*′. In subgraph nesting, the top subgraph is used as the input to the next iteration of Correlated PageRank. We expect the nesting approach to be a more suitable way to decrease subgraph size. In contrast to the threshold approach, the nesting approach iteratively applies the same Correlated PageRank algorithm to subsequent subgraphs; correlation to the phenotype is considered throughout the process, and smaller subgraphs are generated in a more informed way. Within the two sub-approaches, we expect subgraph nesting to perform better, as it continues searching for stronger subgraphs within an already known strong subgraph.

### Correlated Louvain

As before, we begin with an overview of the traditional Louvain algorithm, then introduce our changes. The Louvain algorithm is a two-phase heuristic graph clustering algorithm, consisting of an optimization phase followed by a merging phase (Blondel et al., 2008). It has been shown to consistently produce high quality subgraphs while remaining scalable to very large graphs.

The algorithm starts with each node in a singleton subgraph. During the optimization phase of the algorithm, the modularity, *Q*, is calculated for the current partitioning of the graph *A*_*ij*_ as shown in Equation 6, Equation 7 and Equation 8 (Newman, 2004). *A*_*ij*_ represents the weight of the edge between nodes *i* and *j* while *c*_*i*_ is the subgraph in which node *i* appears.


(6) $$\begin{array}{l} Q=\frac{1}{2m}\underset{i,\;j}{\sum }\left[A_{ij}-\frac{k_{i}k_{j}}{2m}\right]\delta \left(c_{i},\;c_{j}\right) \end{array}$$

(7) $$\begin{array}{l} k_{i}=\underset{j}{\sum }A_{ij} \end{array}$$

(8) $$\begin{array}{l} \delta \;\left(u,\;v\right)=\;\{\begin{array}{l} 1\\ 0 \end{array}\;\frac{if\;u=v}{otherwise} \end{array}$$

Each node is then iteratively considered to be moved to a neighboring subgraph, and the modularity is calculated with the prospective partitioning. The node is moved to the subgraph which yields the largest positive change in modularity, or otherwise remains in its original subgraph if no potential moves yield a positive change. This process is repeated with all nodes iteratively until there is little change in modularity during an iteration.

Once all nodes are moved to the subgraph which yields the largest positive change in modularity, the merging phase begins, and all nodes in a subgraph are merged into one node. New edges are created such that all edges connecting nodes in a subgraph become a self-edge, and all edges connecting nodes in different subgraphs become edges connecting the new merged nodes. The new edges are given a weight equal to the sum of the weights of the edges which merged to form the edge.

The objective function in this algorithm is modularity (Equation 6), which is a measure of connectivity within a subgraph compared to the expected connectivity if all edges were randomly assigned. Similar to conductance, which was used in the Correlated PageRank method, it aims to capture connectivity within subgraphs. By optimizing for connectivity, the Louvain algorithm optimizes subgraphs such that nodes are well connected within a subgraph, and less connected between other subgraphs, effectively finding connected communities within a network.

To optimize this algorithm for an omics dataset, where both connectivity and correlation to a phenotype are important, Correlated Louvain uses the weighted sum of modularity, Q (Equation 6), and Pearson correlation, ρ (Equation 1) in a hybrid objective function. We define *k*_*L*_, a weight parameter to control the effect of modularity and correlation in the hybrid objective function, such that *k*_*L*_ ≤ 1. The hybrid objective function, Equation 9, allows the algorithm to optimize subgraphs such that they are well connected (high modularity) and well correlated to the phenotype (high omics-phenotype correlation). Different weights were tested using a grid search method to find the optimal balance between the quality metrics.


(9) $$\begin{array}{l} Q+\rho =k_{L}*Q+\left(1-k_{L}\right)*\rho \; \end{array}$$

As the Correlated Louvain algorithm produces a partitioning of the graph, which consists of a set of subgraphs, a separate subgraph identification step is not necessary with this method, unlike Correlated PageRank. During our testing, subgraphs produced by this method were small in size, and thus pruning was also not necessary, but would follow a similar procedure to those outlined in Section Subgraph Pruning. The expected size of a significant subnetwork depends heavily on application; in our case study this was informed by the biological processes and literature surrounding COPD. While subgraphs in our tests were not large enough to necessitate further pruning, this may not always be the case with every application.

### Hybrid Approach

A hybrid approach utilizing both Correlated Louvain and Correlated PageRank to generate significant subgraphs was explored. The Correlated Louvain approach described in Section Correlated Louvain, is first utilized to generate a partitioning for the global graph generated by SmCCNet. Correlation to the phenotype is calculated for all non-singleton subgraphs, and the subgraph with the highest correlation is used to seed the Correlated PageRank algorithm. Teleportation values for the nodes in the seed set are weighted by their relative contribution to the subgraph's correlation, as shown in Equation 10. The contribution of node *i* is defined as the difference between the correlation of the subgraph containing the node *i*, *S*, and the subgraph excluding the node *i*, *S'*, and its relative contribution α_*i*_ is the ratio of its contribution to that of the node with the largest contribution, α_max_.


(10) $$\begin{array}{l} S^{\prime }=S\backslash i\\ \rho_{i}=|\rho \left(S\right)|-|\rho \left(S^{\prime }\right)|\\ \alpha_{i}=\frac{\rho_{i}}{\max\left\{\rho_{i^{\prime }}\;|i^{\prime }\in S\right\}}*\alpha_{\max} \end{array}$$

**Figure 1:**
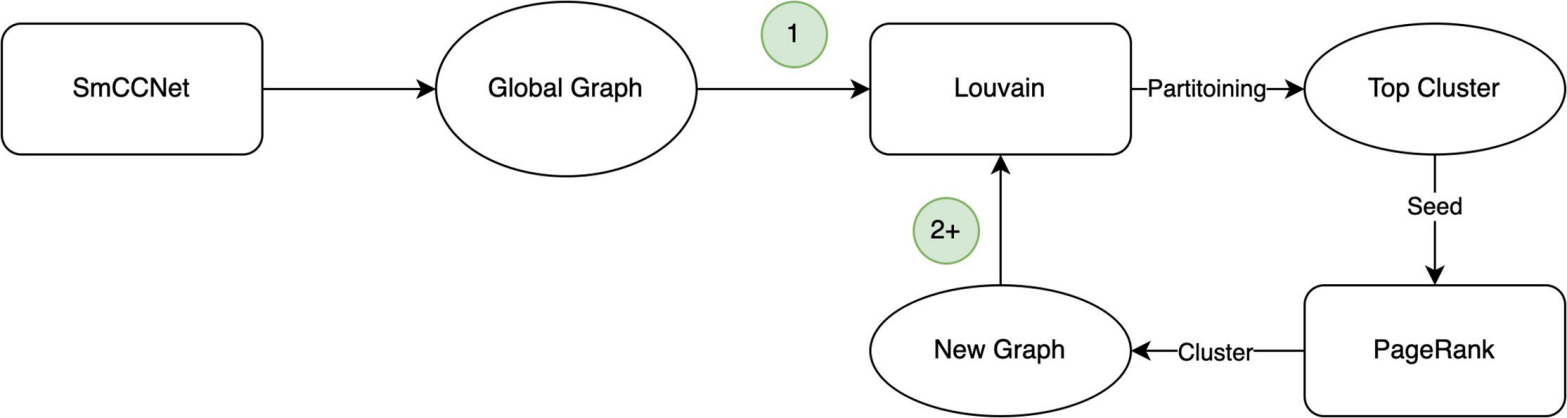
Hybrid clustering procedure. The first iteration (1) uses the global graph provided by SmCCNet, with subsequent iterations (2+) using the cluster generated by PageRank in its place.

The Correlated PageRank algorithm produces a top subgraph, which is then partitioned again using Correlated Louvain. The cycle, depicted in Figure 1, repeats until the size of the subgraph generated by the Correlated PageRank algorithm stops changing, or until it produces a singleton subgraph which cannot be further partitioned by the Correlated Louvain algorithm.

By using the Correlated Louvain algorithm to generate candidate seed nodes for the Correlated PageRank algorithm, the need to pre-select a seed node, whether randomly or based on biological intuition, is bypassed, allowing for a more systematic selection of a good set of seed nodes. This, in turn, should allow the Correlated PageRank algorithm to perform better. Repeating this process has a similar outcome to nesting and pruning, in that it iteratively decreases the size of the network such that the remaining nodes generate strongly correlated and well-connected subgraphs of an appropriate size.

### Subgraph Comparison

Subgraph identified by the above approaches may have significant overlap. Identifying the unique subgraphs from a pool of significant subgraphs is an important task when considering the problem of identifying pathways from a multi-omics network. We developed a method to visually compare two subgraphs, allowing for easy identification of the similarity of two subgraphs, which we discuss below.

Two measures were used to assess the similarity of two subgraphs, computed using the edit distance, or Levenstein distance of node *i*, as shown in Equation 11, where *E*(*i*) represents the set of all unique edges in the graphs G_1_ and G_2_- connected to node n, *d*(*e*) represents the edit distance between edge *e* in G_1_ and G_2_, and *w*(*G*_*i*_, *e*) is the weight of edge e in graph G_i_. Edit distance quantifies the similarity between two graphs by counting the minimum number of edits needed to make the two graphs match; a higher edit distance corresponds to more edits and lower similarity, whereas an edit distance of 0 signifies identical graphs.


(11) $$\begin{array}{l} lev\left(i\right)=\{\begin{array}{l} \infty \;if\;n\notin G_{1}\cup n\notin G_{2}\;\\ \sum_{e\in E\left(i\right)}d\left(e\right)\;otherwise \end{array}\\ d\left(e\right)=\{\begin{array}{c} 1\;if\;\left[e\notin G_{1}\cup e\notin G_{2}\right]\cup \left[w\left(G_{1},\;e\right)\neq w\left(G_{2},\;e\right)\right]\\ 0\;otherwise \end{array} \end{array}$$

**Figure 2:**
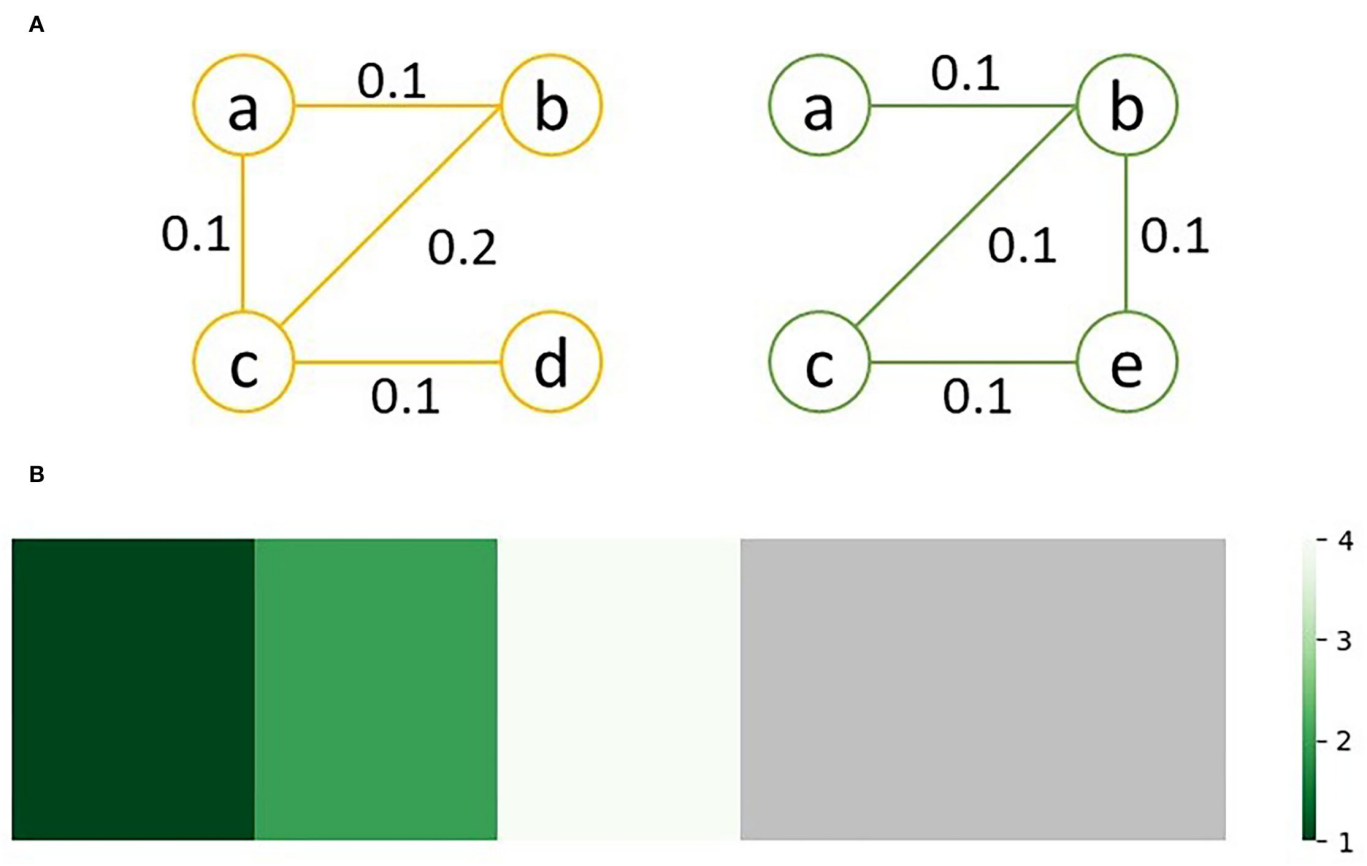
Graph comparison between **(A)** two sample graphs, G_1_ (left) and G_2_ (right); **(B)** shows the heatmap visualization of the edit distances between G_1_ and G_2_, with the green regions representing similarity between he graphs and gray regions representing differences; the ration between the regions visually represents how similar the two graphs are.

As an example, consider the sample graphs G_1_ (left) and G_2_ (right) in Figure 2A. Since nodes d and e don't exist in both graphs, *lev*(*d*) = *lev*(*e*) = ∞. Looking at node c, which exists in both graphs, *lev*(*v*) = 4; of the 4 unique edges connected to c in either graph, one edge, bc, has a different weight, and three edges, ac, cd and ce, only exist in one graph. Similarly, *lev*(*a*) = 1, since edge ab exists in both graphs with equal weight while edge ac only exists in G_2_. The sorted edit distance vector for the sample graphs is as follows:


$$\begin{array}{l} \vec{\begin{array}{ll} \begin{array}{lll} \begin{array}{l} a\\ 1 \end{array} & \begin{array}{l} b\\ 2 \end{array} & \begin{array}{l} c\\ 4 \end{array} \end{array} & \begin{array}{ll} \begin{array}{l} d\\ \infty \end{array} & \begin{array}{l} e\\ \infty \end{array} \end{array} \end{array}} \end{array}$$

This can be visualized as a heatmap to quickly assess and compare the similarity of multiple pairs of graphs. For the above sample, the heatmap shown in Figure 2B is generated.

We also assess similarity using the Jaccard index, J, as shown below in Equation 12. The Jaccard index is the ratio of the intersections of two graphs, G_1_ and G_2_ to their union, and is a quantitative measure that can be used to assess how similar or dissimilar they are. A higher Jaccard index implies higher similarity.


(12) $$\begin{array}{l} J\left(G_{1},\;G_{2}\right)=\frac{\left|G_{1}\cap G_{2}\right|}{\left|G_{1}\cup G_{2}\right|} \end{array}$$

Graph similarity is primarily used to find unique subgraphs; the Correlated PageRank method will generate different subgraphs given different seed nodes, but some of these subgraphs may be very similar. Similar subgraphs are not informative as they uncover the same biological pathways. To find unique subgraphs, and thus unique pathways, different highly correlated subgraphs are compared, and those showcasing significant dissimilarity are selected for further analysis. Significant dissimilarity is discovered by plotting the Jaccard indices and visually identifying a steep drop in the index values indicating a significantly dissimilar subgraph.

### Platform

Correlated PageRank was implemented using Python 3.8. Correlated Louvain was implemented in C++ and exposed to Python as a module. All testing was done using Python 3.8. The graph used in our case study was generated using the SmCCNet R package, version 0.99.0, and exported to be used with the NetworkX and igraph Python packages during testing. Tests were conducted on an Intel Core i9-11900K. Code for our proposed methods is available on GitHub at bdlab-ucd/correlated-louvain. The datasets used in our case study, Section Experimental Evaluation: A Case Study, is available from COPDGene; while it is a public dataset, it is made available by request with a submitted proposal detailing the intended usage of the dataset.

## Experimental Evaluation: a Case Study

To study the behavior of our proposed techniques and compare them to the existing hierarchical clustering approach, we used a COPD multi-omics dataset as a model. We use protein and metabolite omics datasets, consisting of 1,317 and 996 biomarkers, respectively. The phenotype (target) dataset consists of FEV_1_% measurements, which is commonly used for COPD diagnosis and severity evaluation. All measurements were taken from 994 subjects common across the two omics datasets and the phenotype dataset. Using the same dataset Mastej et al. reported two omics subnetworks, one with 13 proteins and 7 metabolites having −0.34 correlation (*p* = 2.5 ^*^ 10^−28^) to lung function, and another with 13 proteins and 10 metabolites having −0.27 correlation (*p* = 2.6 ^*^ 10^−17^) to percent emphysema (Mastej et al., 2020).

### Experimental Methodology

For each of the approaches we introduced, we first conducted behavioral tests to optimize each approach individually. The top subgraphs produced by each approach, as well as those produced by the current hierarchical approach, were compared. The results reported below were produced by running the experiment once for each set of parameters.

All tests were run using the same protein-metabolite omics dataset and FEV_1_% phenotype, for the same set of subjects, as described above. SmCCNet weights, which control the weight of different features when calculating canonical correlation during the SmCCNet algorithm, represented as *a-b-c*, were explored using the Correlated PageRank approach, detailed below, and the optimal set of weights was then used to generate the same graph used in all final comparisons.

Our primary measure of subgraph quality was correlation to the phenotype, ρ (Equation 1), with a higher absolute correlation indicating a more significant subgraph which better describes the phenotype. We also report conductance, Φ (Equation 4), to summarize connectivity within each subgraph relative to the global network. Subgraph size is also reported, which can be an indication of subgraphs which are likely too small or too large to be of importance. While appropriate subgraph size will depend on the specific omics used and intended application, subgraphs with hundreds of nodes are likely too broad to be informative, and subgraphs with very few nodes are potentially too narrow in scope and ignore important participants in the pathway they describe.

### Experimental Results

For each of the three proposed approaches, different parameters needed to be tuned to find the optimal subgraphs, the results of which are discussed below.

#### Correlated PageRank

Correlated PageRank is affected by factors such as tolerance (ϵ), teleportation factor (α), the selected seed node, and the weights used in the SmCCNet algorithm to generate the global graph. Tolerance, teleportation factor, and SmCCNet weights were studied using a grid search method, testing different values to find an optimal set of parameters. Seed nodes were selected at random to study their influence on the resulting subgraphs' correlation and conductance.

Correlated PageRank consisted of two similar but distinct approaches, sequential and simultaneous as discussed in Section Subgraph Identification, which are discussed separately in Sections Subgraph Identification–Sequential Approach and Subgraph Identification–Simultaneous Approach. The size of subgraphs identified by Correlated PageRank necessitate pruning, which is discussed in Section Subgraph Pruning.

##### Subgraph Identification–Sequential Approach

The effects of tolerance (ϵ) on the Correlated PageRank subgraph size and conductance are shown in Table 1. ϵ has a considerable role on the size of the subgraphs. When ϵ < 1^*^10- 2, the subgraphs generated were large (419 nodes) and the absolute correlation was small (0.14), whereas when ϵ > 1^*^0^−3^, the tolerance stops affecting size and correlation significantly. As mentioned before, the PageRank search continues until the difference between PageRank scores in two consecutive iterations is below ϵ. When ϵ is lowered, the random walker can visit more nodes on the graph, and thus subgraph size and correlation increase.

**Table 1:** Tolerance effect on Correlated PageRank for 1-10-10 graph at α = 0.04 and seed = 2,137.

**ϵ**	**Subgraph size**	**Φ**	**ρ (*p*_value)**	**Φ + ρ**
**Sequential Correlated PageRank (1-10-10)**				
1.00e-01	419	0.502	−0.14 (5.2e-06)	
1.00e-02	419	0.502	−0.14 (5.2e-06)	
1.00e-03	337	0.502	−0.3 (9.76e-22)	
1.00e-04	337	0.502	−0.3 (9.76e-22)	
1.00e-05	322	0.501	−0.29 (1.69e-20)	
1.00e-06	322	0.501	−0.29 (1.69e-20)	
**Simultaneous Correlated PageRank (1-10-10)**				
1.00e-01	439	0.547	−0.17 (1.29e-07)	0.098
1.00e-02	439	0.547	−0.17 (1.29e-07)	0.098
1.00e-03	328	0.528	−0.31 (7.43e-23)	0.226
1.00e-04	328	0.528	−0.31 (7.43e-23)	0.226
1.00e-05	303	0.552	−0.3 (1.73e-21)	0.215
1.00e-06	303	0.552	−0.3 (1.73e-21)	0.215

Table 2 displays the Correlated PageRank results of the 1-10-10 graph for different teleportation (α) values. As the α increases, the subgraph sizes decrease, and the correlation experiences very little change. Subgraph size decreases substantially slower once α > 0.04. When α increases, the random walker is more likely to return to the seed node with each step, and is thus less likely to visit neighboring nodes and traverse deeper into the graph. The correlation values are quite high at 0.3 (*p* = 7.93 ^*^ 10^−21^), which is about 1.5 times higher than the previous hierarchical results with the same graph (-0.18, *p* = 2.3 ^*^ 10-^−8^).

**Table 2:** Teleportation effect on Correlated PageRank for 1-10-10 graph at ε = 1.0e-4 and seed = 2,137.

**α**	**Subgraph size**	**Φ**	**ρ (*p*_value)**	**Φ + ρ**
**Sequential Correlated PageRank (1-10-10)**				
0	425	0.501	0.29 (2.72e-20)	
0.02	352	0.501	−0.3 (1.72e-22)	
0.04	337	0.502	−0.3 (9.76e-22)	
0.06	329	0.501	−0.29 (7.39e-21)	
0.08	328	0.502	−0.29 (7.93e-21)	
0.1	325	0.503	−0.29 (7.69e-21)	
**Simultaneous Correlated PageRank (1-10-10)**				
0	453	0.59	0.31 (6.31e-23)	0.22
0.02	350	0.505	−0.3 (9.63e-23)	0.22
0.04	328	0.528	−0.31 (7.43e-23)	0.226
0.06	324	0.513	−0.3 (1.79e-21)	0.219
0.08	319	0.524	−0.3 (6.89e-22)	0.218
0.1	318	0.524	−0.3 (7.32e-22)	0.218

**Figure 3:**
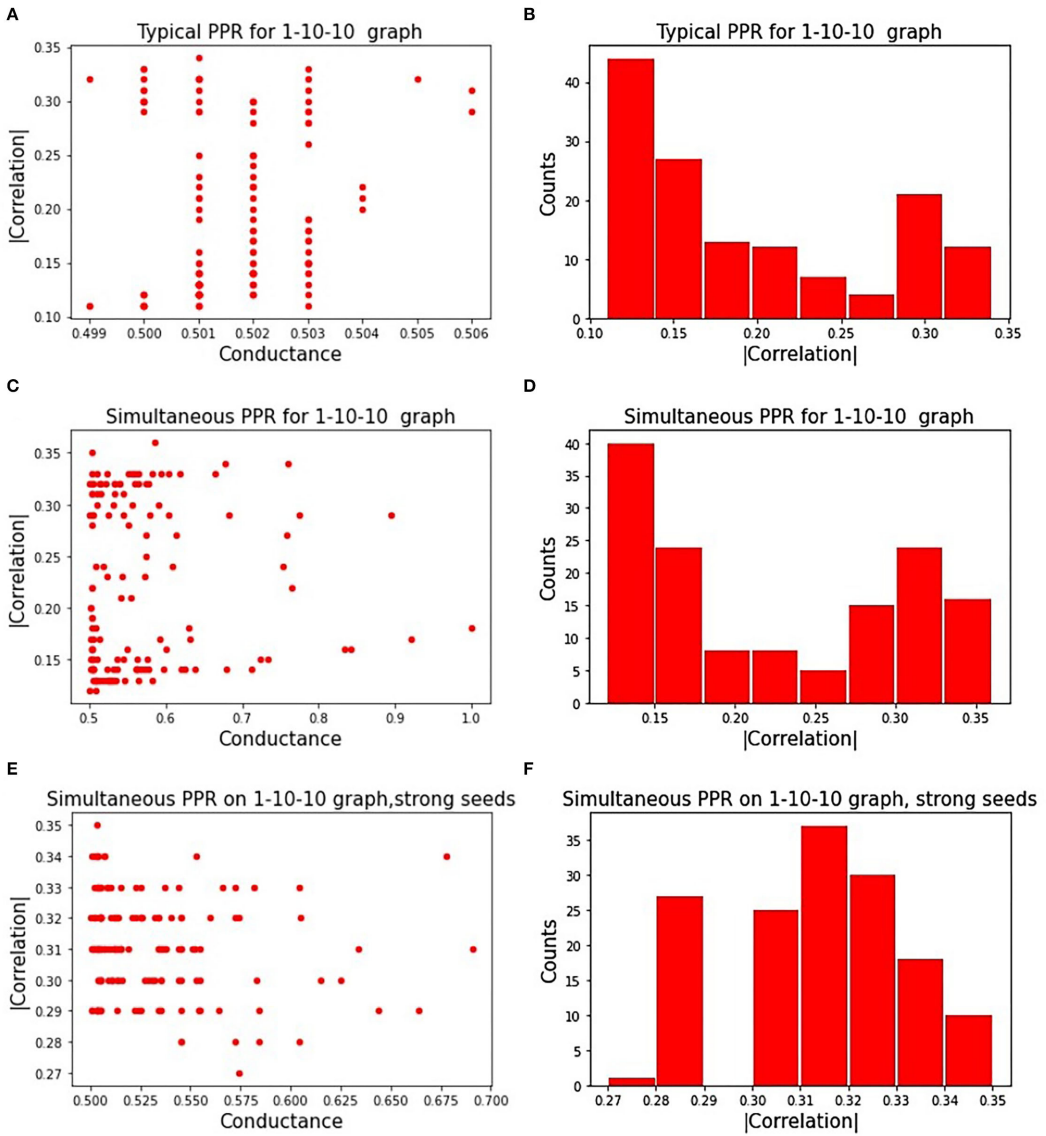
Correlated PageRank results: **(A)** shows the randomized search results for the sequential approach to Correlated PageRank; **(B)** shows a distribution of |ρ| for subgraphs identified by sequential Correlated PageRank with randomized seed selection; **(C)** shows randomized search results for the simultaneous approach to Correlated PageRank; **(D)** shows a distribution of |ρ| for subgraphs identified by simultaneous Correlated PageRank with randomized seed selection; **(E)** shows randomized search results for simultaneous Correlated PageRank with selected strong seeds only; **(F)** shows a distribution of |ρ| for simultaneous Correlated PageRank with strong seeds.

Figure 3A displays the influence of seeds on a subgraph's correlation and conductance. Absolute correlation ranged from 0.1 to 0.35, while conductance remained near 0.5 for all chosen seeds. Figure 3B shows the distribution of absolute correlation with the phenotype, with a majority of the correlation values falling below 0.2. This shows the importance of the seed on the quality of the resulting subgraph, highlighting one of the limitations of a PageRank based approach.

Appendix A shows the results from sequential Correlated PageRank at various SmCCNet weights. These experiments were conducted with ϵ in the range of 1 ^*^ 10^−1^-1 ^*^ 10^−8^ and α in the range of 0–0.15. The absolute correlations of the subgraphs produced from the 1-10-10 graph were about twice those of the 1-5-5 subgraphs, with conductance being about a third. SmCCNet weights are applied to the canonical correlation to adjust the relationship between different features. Larger *b* and *c* values create a stronger correlation between each of the omics and the phenotype compared to the correlation between the two omics. This has the effect of pruning the weak edges in the global graph generated by SmCCNet, resulting in stronger edge weights overall, and higher quality subgraphs when Correlated PageRank is applied. This experiment was also conducted with SmCCNet weights of 1-11-11 and 1-1-1, both of which resulted in small global networks not suitable for subgraph identification, at 32 and 228 nodes, respectively. SmCCNet weight, as seen here, can have a significant effect on the subgraphs found downstream. The 1-10-10 set of weights produced the most favorable results, so it was used for all further experiments.

##### Subgraph Identification–Simultaneous Approach

Table 1 summarizes the effect of tolerance (ϵ) on the size of the subgraphs generated with the simultaneous Correlated PageRank approach using Troponin (node 2,137) as a seed. As ϵ decreases, the size of the subgraphs and the conductance decrease while the absolute correlation and Φ + ρ increase. At a lower tolerance, the random walker spends longer traversing nodes and as a result visits high degree nodes more often, resulting in a smaller subgraph and larger correlation. Although not shown, similar results were seen with other seed nodes.

Table 2 shows the influence of teleportation probability (α) on the size of simultaneous Correlated PageRank subgraphs. We see that a larger α results in a smaller subgraph and higher conductance, while the correlation and Φ + ρ see little change. These are similar results to what was seen with the sequential Correlated PageRank method.

Figure 3C displays the correlation and conductance of subgraphs generated by simultaneous Correlated PageRank using a random selection of seed nodes. The experiment conditions are the same as those used with the sequential Correlated PageRank approach. As expected, seeds have a significant effect on the correlation and conductance values. Compared to the sequential Correlated PageRank approach, we see a wider range of conductance values, ranging from 0.5 to 1, instead of all being roughly 0.5. Figure 3D shows the distribution of absolute correlation values. We again see a majority of subgraphs with correlation lower than 0.2, but there is a noticeable increase in subgraphs with correlation higher than 0.3, indicating better performance compared to the sequential approach.

We repeated these experiments with only strong seeds, shown in Figures 3E,F. Strong seeds were selected by applying Correlated PageRank on the full graph with parameters α = 0.95, ϵ = 5.0^*^10^−4^, then selecting seed nodes from subgraphs with |ρ| > 0.33. These seeds produced subgraphs with stronger correlation and lower conductance, as expected.

Appendix A shows the effect of different SmCCNet weights on the simultaneous Correlated PageRank subgraphs. The 1-10-10 subgraphs have higher correlation and lower conductance compared to the 1-5-5, similar to the results seen with the sequential approach.

##### Subgraph Pruning

Two pruning approaches were tested, with the goal of reducing subgraph size while maintaining quality. Results from these approaches under different conditions are summarized below.

Table 3 summarizes the threshold pruning results for the subgraphs generated by the seed 248. A higher threshold resulted in a smaller subgraph with lower correlation and more balanced omics ratio. Mastej et al. applied the same approach to remove weak edges from hierarchical subgraphs (Mastej et al., 2020). In their study, pruning resulted in a balanced protein-metabolite ratio without significantly affecting correlation. A potential cause for the decrease in correlation when threshold pruning is applied to Correlated PageRank subgraphs is that it may already be accounting for weak edges when generating scores, thus further pruning leads to a decrease in quality.

**Table 3:** Threshold pruning on the subgraph created by seed = 248.

**Prune threshold**	**Remaining edges (%)**	**Subgraph size**	**Prot_ratio**	**ρ (*p*-value)**
0	0	249	0.65	−0.33 (2.48e-27)
0.01	45.3	216	0.65	−0.32 (1.99e-25)
0.02	59.2	211	0.65	−0.32 (1.25e-25)
0.03	68.2	200	0.65	−0.32 (2.41e-25)
0.04	74.8	189	0.66	−0.32 (1.43e-24)
0.05	79.3	179	0.66	−0.31 (3.6e-24)
0.06	83.1	168	0.66	−0.31 (1.4e-23)
0.07	86.1	157	0.65	−0.31 (2.87e-23)
0.08	88.5	150	0.66	−0.3 (9.08e-23)
0.09	90.5	143	0.65	−0.3 (1.74e-22)

Two nesting approaches were investigated, global and subgraph nesting. Global nesting consists of removing the top subgraph from the global graph, then applying Correlated PageRank to the remaining graph. Subgraph nesting instead isolates the top subgraph and applies Correlated PageRank to it instead of the global network. To obtain high correlation subgraphs, strong seeds were chosen at each iteration.

Table 4 summarizes the global nesting result. Subgraph size and correlation decreased, and conductance increased with each iteration of global nesting. This is likely due to the removal of the top subgraph from the global graph leaving a residual graph with weaker edges and less correlated nodes. As strong subgraphs are removed, Correlated PageRank produces lower quality subgraphs from the remainder of the graph.

**Table 4:** Results of graph and subgraph nesting.

**α**	**ϵ**	**Seed**	**Subgraph size**	**Φ**	**ρ (*p*-value)**	**Φ + ρ**
**Graph nesting (seed: 248)**						
0.05	0.0005	1,502	277	0.553	−0.34 (1.41e-27)	−0.251
0.1	0.0002	1,805	101	0.664	−0.26 (5.36e-17)	−0.168
0.1	0.001	1,867	68	0.673	−0.24 (2.02e-14)	−0.149
0.1	0.0003	893	32	0.728	−0.16 (8.15e-07)	−0.071
**Subgraph nesting (seed: 1502)**						
0.15	0.00056	707	177	0.611	−0.35 (3.44e-29)	−0.254
0.01	0.00078	2,193	54	0.524	0.32 (9.29e-25)	−0.236

Table 4 also summarizes the results of subgraph nesting, using the top subgraph from the first iteration of global nesting as the starting graph. We again see a decrease in subgraph size and correlation. Despite this, subgraphs produced by subgraph nesting display higher correlation and lower conductance when compared to those produced by global nesting. Subgraph nesting likely produces higher quality subgraphs since it uses a high-quality subgraph as its starting point, compared to using the lower quality residual of the global network in global nesting.

#### Correlated Louvain

The Correlated Louvain method is affected by the weight used in the objective function *k*_*L*_, which we studied using a grid search. Additionally, as Correlated Louvain is a global clustering approach, we studied the distribution of correlations of the clusters generated by the algorithm to better understand the quality of clusters generated by the algorithm. Finally, we studied the behavior of the algorithm at different levels in the hierarchy to understand how the partitionings generated evolve as the algorithm progresses.

The primary parameters to adjust in the Louvain algorithm implementation were the weights used in the objective function, controlling the balance between modularity and correlation in the produced subgraphs. A grid search was applied to a range of weights, the results of which are summarized in Table 5. The best subgraphs, in terms of correlation, were produced when *k*_*L*_ was set to 0.2, which produced a top subgraph with ρ = −0.29 (*p* = 1.28 ^*^ 10^−20^). We see, however, that all subgraphs produced are much smaller than those produced by the Correlated PageRank method. While this forgoes the necessity to prune the resulting subgraphs, it also may heavily limit the useful information which can be extracted from this subgraph. The largest subgraph produced was of size 39, but correlation drops to 0.24 (*p* = 4.23 ^*^ 10^−14^).

**Table 5:** Correlation with different modularity and correlation weights.

**k_**L**_**	**Subgraph size**	**Φ**	**ρ (*p*-value)**
0.8	39	0.94	0.24 (4.23e-14)
0.6	7	0.99	0.26 (1.29e-16)
0.4	7	0.99	0.22 (1.35e-12)
0.2	6	0.99	−0.29 (1.28e-20)
0	6	0.99	0.25 (3.70e-15)

Across all tests, we see alarmingly high conductance. However, we see an increase in conductance which correlates with the increase in network size; the largest subgraph, generated by *k*_*L*_ = 0.8, also has the lowest conductance at 0.94, while all other weights generate subgraphs with 0.99 conductance and <10 nodes. This also extends to the Correlated PageRank results, where much larger subgraphs result in significantly lower conductance. The size of subgraphs is likely the key contributing factor the high conductance; since the global network consists of many subgraphs, and connections within a small subgraphs are inherently limited, conductance may highlight weak inter-subgraph connections due to the sheer number of subgraphs in comparison to the small number of intra-subgraph connections. We don't believe this affects subgraph quality, as conductance is only used for comparison between methods and does not affect the algorithm's performance.

**Figure 4:**
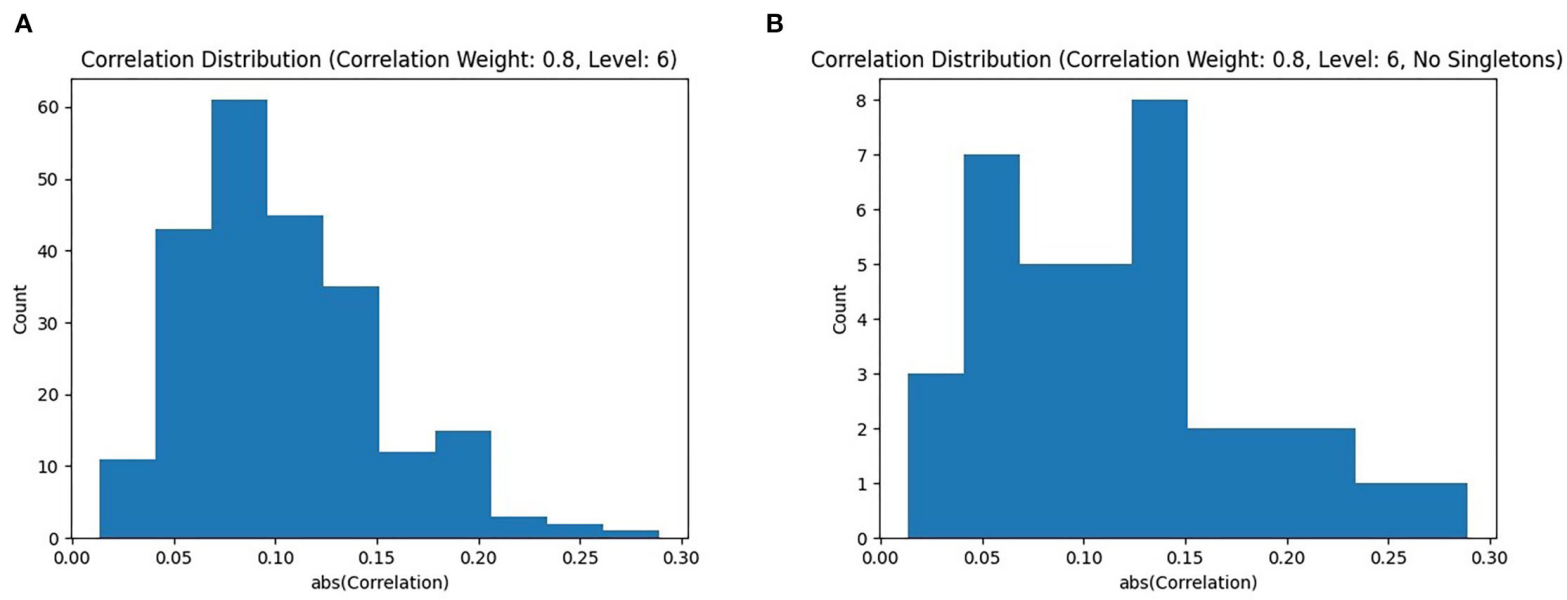
Correlated Louvain results: **(A)** shows a distribution of |ρ| produced by the Correlated Louvain method with *k*_4_ = 0.8; **(B)** shows the same distribution but with singleton clusters removed, only showing subgraphs with |*V*| ≥ 2.

The distribution of absolute correlations, shown in Figure 4A, shows a roughly normal distribution, with the majority of correlation values falling below 0.2. However, since the Louvain algorithm is a global clustering algorithm, and thus places every node in a subgraph, some subgraphs are singletons. The distribution with singletons removed is shown in Figure 4B. The correlation values follow a similar pattern, with a majority of subgraphs still under a correlation of 0.2, but we can see a few higher quality subgraphs. It is evident that the majority of singleton subgraphs are less correlated to the phenotype, as their removal results in a dramatic drop in the number of subgraphs with |ρ| <0.2.

**Figure 5:**
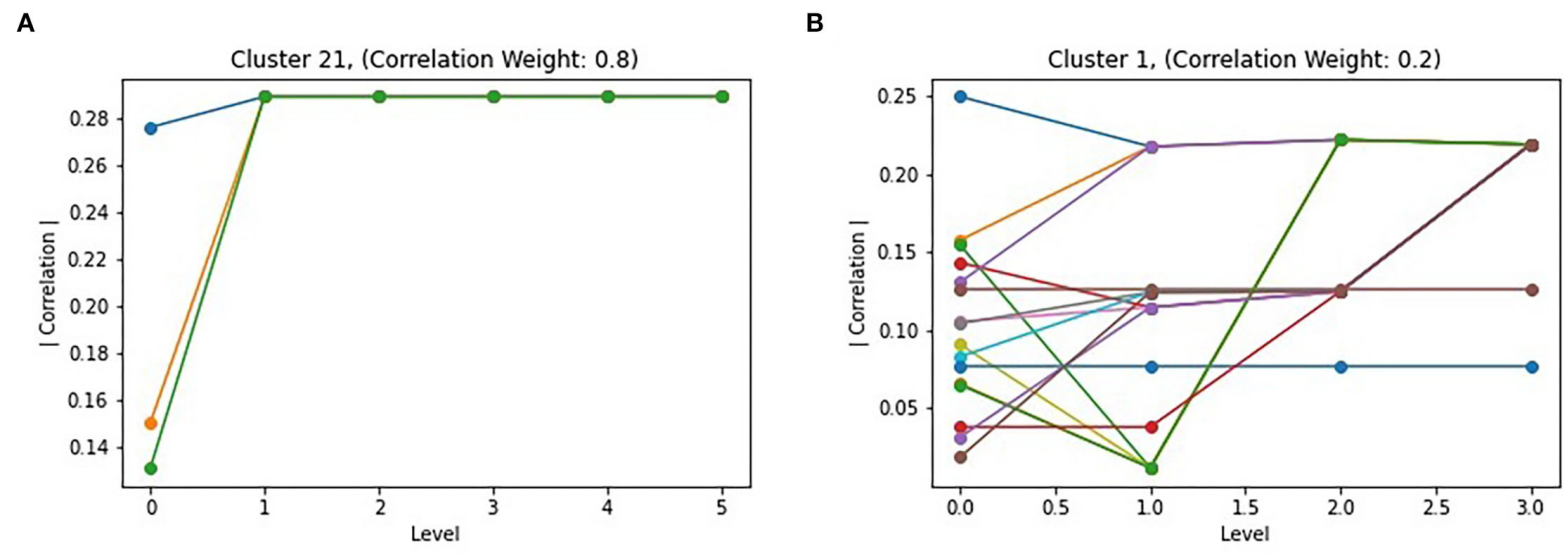
Level effect of Correlated Louvain: **(A)** shows the intermediate clustering of cluster 21 with *k*_4_ = 0.8, representing the common behavior of intermediate clusters; **(B)** shows uncommon behavior or intermediate clusters, such as cluster 1 with *k*_4_ = 0.2.

The Louvain algorithm, similarly to other hierarchical approaches, generates graph partitions at multiple levels, which each iteration of the two phases generating a new level in the hierarchy. While the final level should provide the optimal partitioning of the graph, and thus the best subgraphs, intermediate levels may be informative. To study the behavior of the algorithm, the correlation of all intermediate subgraphs that merged to form a subgraph in the final partitioning were plotted. Two examples are shown in Figure 5. For the majority of the top subgraphs, they behave similar to subgraph 21 in Figure 5A, with nodes merging into a single subgraph by the second iteration of the algorithm, with a consistent increase in absolute correlation when doing so. However, Figure 5B shows that this is not always the case, with a subgraph following a less direct path toward the optimal partitioning. Notably, this behavior occurred when the correlation portion of the objective function was weighted less (k_L_ = 0.8), which is also the partitioning which resulted in larger but less correlated top subgraphs. This is possibly due to the larger effect modularity has outcompeting correlation, resulting in merges which sometimes reduced correlation, but likely lead to a more interconnected subgraph.

#### Hybrid Approach

As we saw in both Correlated PageRank approaches, the teleportation factor has little effect on correlation, primarily affecting subgraph size. However, we see that for α values <0.04, the effects of teleportation have a less pronounced effect. As such, an α value of 0.04 was selected for these tests. Similarly, a tolerance factor of 1^*^10^−6^ was used for all trials. Different weight values for the Correlated Louvain algorithm, k_L_, were tested.

A grid search technique similar to that used with the Correlated Louvain algorithm was used to test the effect of weight on the hybrid approach. The effect of different weights on the most correlated subgraph produced during the Correlated PageRank step is summarized in Table 6. We see high correlation values for all weight values, with all subgraphs identified outperforming the traditional hierarchical method and the Correlated Louvain algorithm and performing equal or better than the Correlated PageRank method. Conductance values are closer to those produced by the Correlated PageRank method, near 0.5, and are much lower than those produced by the Correlated Louvain algorithm, which implies more well-connected subgraphs. Subgraph sizes are much smaller than those produced by Correlated PageRank, with the exception of a 317-node subgraph produced when *k*_*L*_ = 0.2, while also being larger than the sub-10-node subgraphs produced by the Correlated Louvain method. Unfortunately, there doesn't appear to be a pattern between weights and correlation, with both low and high k_L_ values producing highly correlated subgraphs of similar size and conductance.

**Table 6:** Effect of Louvain weights on top subgraphs produced by the hybrid approach.

**k_**L**_**	**Subgraph size**	**Φ**	**ρ (*p*-value)**
0.8	25	0.746	0.41 (1.19e-41)
0.6	69	0.514	−0.35 (2.75e-29)
0.4	39	0.515	−0.34 (1.45e-27)
0.2	317	0.503	−0.33 (1.26e-26)
0.2	23	0.552	0.33 (7.58e-27)
0	22	0.548	−0.39 (2.31e-37)
0	5	0.592	−0.39 (5.39e-38)
0	4	1	−0.39 (8.03e-37)

**Figure 6:**
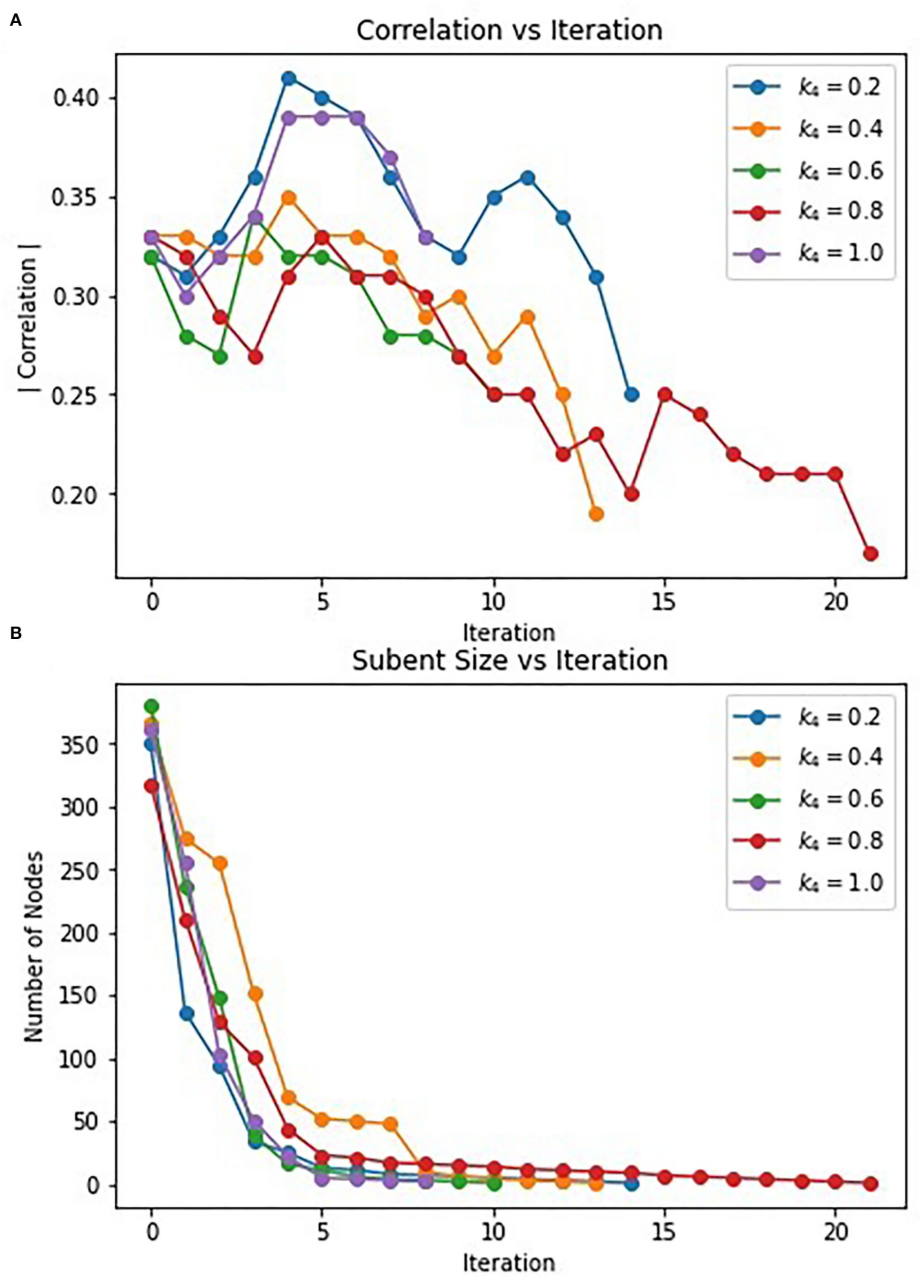
Change in correlation **(A)** and subnet size **(B)** of subnets produced by PageRank in each iteration.

**The hybrid approach** terminates when the subgraphs produced by the Correlated PageRank step stop changing in size. However, the most correlated subgraphs were produced during intermediate steps. Figure 6 displays the change in correlation and subgraph size of subgraphs produced by the Correlated PageRank step in each iteration. Correlation generally increases during the first few iterations of the procedure, reaching a peak at iteration 3–5 before beginning to decrease. Subgraphs rapidly decrease in size, with a visible elbow corresponding to the decrease in correlation. The algorithm seems to favor always decreasing subgraph size, leading to a decrease in subgraph quality, likely due to a constriction in the available nodes in the network. Once the most correlated subgraph is found, continued restriction on the network size leads to important nodes being removed and a decrease in overall correlation.

#### Comparative Analysis

Table 7 contains a summary of the top subgraphs generated by Correlated PageRank, Correlated Louvain, hybrid approach, and the traditional hierarchical approaches. As can be seen, with the 1-10-10 SmCCNet weights, both Correlated PageRank and Correlated Louvain outperform the hierarchical approach, with much higher correlation to the phenotype. Both Correlated PageRank approaches generate much larger subgraphs, which can be pruned while still maintaining a higher correlation value. The Correlated Louvain approach, however, generates much smaller subgraphs overall, with the exception of the subgraphs produced when k_L_ = 0.8 which produced a more comparably sized subgraph while still maintaining a higher correlation.

**Table 7:** Summary of top subgraphs.

**α**	**ϵ**	**Seed**	**k_**L**_**	**Subgraph size**	**Φ**	**ρ (*p*-value)**	**Φ+Φ**
**Sequential Correlated PageRank (1-10-10)**							
0.03	1.00E-04	2,137		341	0.5	−0.31 (6.18e-23)	
0.08	1.00E-04	303		321	0.5	−0.31 (2.68e-23)	
0.01	1.00E-03	905		307	0.5	−0.29 (8.79e-21)	
**Simultaneous Correlated PageRank (1-10-10)**							
0.04	1.00E-04	2,137		328	0.53	−0.31 (7.43e-23)	0.23
0.01	1.00E-03	303		385	0.55	0.32 (2.18e-24)	0.23
0.1	1.00E-06	905		208	0.63	−0.34 (2.50e-28)	0.24
**Hierarchical clustering (1-10-10)**							
				29		0.18 (1.90e-08)	
**Hierarchical clustering (1-11-11)**							
				22		0.33 (3.90e-26)	
**Correlated Louvain (1-10-10)**							
			0.8	39	0.94	0.24 (4.23e-14)	1.18
			0.6	7	0.99	0.26 (1.29e-16)	1.25
			0.4	7	0.99	0.22 (1.35e-12)	1.21
			0.2	6	0.99	−0.29 (1.28e-20)	0.70
			0	6	0.99	0.25 (3.70e-15)	1.24
**Hybrid approach (1-10-10)**							
0.04	1.00E-06		0.8	25	0.75	0.41 (1.19e-41)	1.16
0.04	1.00E-06		0.6	69	0.51	−0.35 (2.75e-29)	0.16
0.04	1.00E-06		0.4	39	0.52	−0.34 (1.45e-27)	0.18
0.04	1.00E-06		0.2	23	0.55	0.33 (7.58e-27)	0.88
0.04	1.00E-06		0	22	0.55	−0.39 (2.31e-37)	0.16

The hybrid approach appears to combine the best properties of both methods, and further improves on them. Correlation values are higher than those produced by the Correlated Louvain method, and similar to or better than those produced by the Correlated PageRank approach. Notably, correlation values produced by the hybrid approach are a significant improvement over those produced by the state of the art hierarchical approach, achieving a maximum |ρ| = 0.44, compared to the maximum |ρ| = 0.33 seen with hierarchical clustering, while maintaining a similarly sized subgraph. Subgraphs produced by this method are much smaller than those produced by the Correlated PageRank approach while not being as restricted in size as those produced by the Correlated Louvain method. They are similarly sized to those produced by the hierarchical approach and are an appropriate size for biological analysis.

### Subgraph Comparison

Using a pool of significant subgraphs identified by the Correlated PageRank approach, we applied our subgraph comparison approach to identify a set of unique subgraphs, which can be used for downstream analysis and applications. The subgraph with the highest correlation to the phenotype, seed node 175 with ρ = 0.35 (*p* = 1.22 ^*^ 10^−29^) was selected as the reference subgraph, and all other subgraphs were compared to it. To find unique subgraphs, the reference subgraph was compared to all other subgraphs, and the resulting Jaccard indices were ranked. High Jaccard indices indicates a subgraph highly similar to the reference subgraph, which was removed from later comparisons, while a sudden drop in Jaccard indices indicated the first significantly different subgraph, which was used as the next reference subgraph. This process was repeated until all top subgraphs exhausted; any subgraphs not considered highly similar to a reference node are sufficiently unique and can be used for further biological analysis.

**Figure 7:**
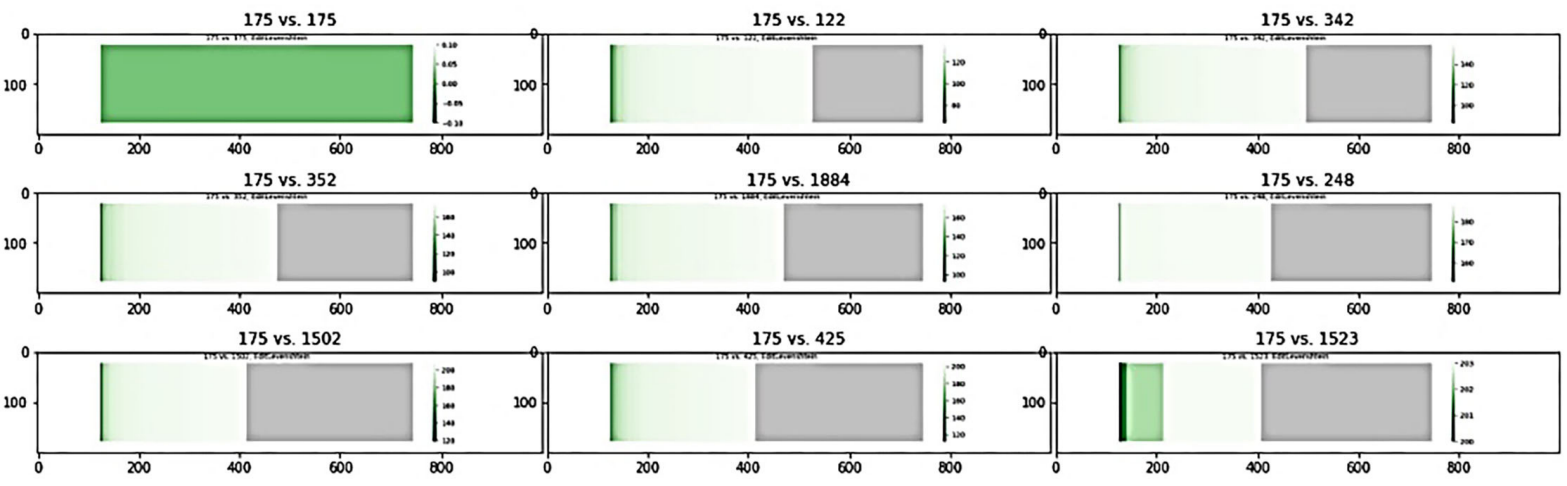
Visual comparison of subnet 175 with other top subnets.

Figure 7 shows a sample of the visual comparisons generated when comparing subgraph 175 with other top subgraphs, and the results are summarized in Table 8. Visually, we observe that subgraphs 122 and 345 are quite similar, whereas 122 and 1,523 are quite different; this is observed as a larger portion of the generated heatmap being green, signifying similarity, than gray, which signifies difference. This observation is supported by the Jaccard indices, with 122 and 345 showing comparable Jaccard values.

**Table 8:** Top simultaneous PageRank subgraphs from visual comparison.

**Subgraph 1**	**Subgraph 2**	**Subgraph Size**	**Φ**	**ρ (*p*-value)**	**J**
175	175	293	0.503	0.35 (1.22e-29)	1
175	122	335	0.503	0.34 (2.52e-28)	0.64
175	352	334	0.504	−0.34 (5.99e-28)	0.56
175	248	252	0.501	−0.34 (1.7e-27)	0.49
175	1,523	246	0.507	−0.34 (1.54e-27)	0.45
122	122	335	0.503	0.34 (2.52e-28)	1
122	342	334	0.502	0.34 (8.46e-28)	0.69
122	425	261	0.678	0.34 (1.7e-27)	0.56
122	248	252	0.501	−0.34 (1.7e-27)	0.45
106	352	334	0.504	−0.34 (5.99e-28)	0.61
106	1,502	277	0.553	−0.34 (1.41e-27)	0.53

## Conclusion and Future Work

In this study, we proposed three novel implementations of significant subgraph identification techniques which can be used to study the relationship between mixed omics datasets and a disease phenotype. The proposed Correlated PageRank approach combines conductivity and correlation to find significant subgraphs, while the Correlated Louvain approach combines modularity and correlation. A hybrid approach, combining the Correlated Louvain and Correlated PageRank methods was also explored, using the Correlated Louvain method to better inform seeds used during the Correlated PageRank algorithm. We discussed the effects of various parameters on each approach's performance. When comparing the subgraphs produced by these approaches, they all outperform the state of the hard hierarchical clustering currently in use, by producing subgraphs with much higher correlation to the phenotype. While this wasn't a direct focus of this study, these approaches should also prove to be much more scalable, which is necessary when applied to large biological datasets.

While we used SmCCNet to generate a protein/metabolite multi-omics network in our case study, these approaches can be used to detect significant subgraphs in a multitude of similar graphs, where correlation to a target variable and topological cohesion define subgraph quality and significance. For example, these approaches can be used to detect significant subgraphs in the single-omics networks generated by WGCNA.

These approaches do highlight some limitations, which will be addressed in future work. Correlated PageRank currently relies on the selection of a good seed, which must be chosen based on node degree, or ideally informed by biology. Correlated Louvain approach suffers from small subgraph size, which limits the informativeness of the subgraphs generated. The hybrid approach appears to address the limitations of both methods but introduces longer runtimes. Future work will focus on addressing these limitations, as well as applying these approaches to other datasets, such as RNA and mRNA omics, or different phenotypes.

## Data Availability Statement

The data analyzed in this study is subject to the following licenses/restrictions: data access restricted to approved institutions/investigators/studies. Requests to access these datasets should be directed to FB-K, farnoush.banaei-kashani@ucdenver.edu.

## Author Contributions

MA-H and MN contributed equally to the subgraph detection methodology. SH contributed to the comparison methodology. KP and RB provided expertise in biological aspects of example application (COPD) and editing manuscript. YZ, KK, LL, RB, and FB-K provided suggestions for improving methodology, formalizing problem, editing manuscript, and evaluating results. WL provided and helped prepare dataset for example application (COPD). All authors contributed to the article and approved the submitted version.

## Funding

This work was supported by the National Institutes of Health (NIH), National Heart, Lung, and Blood Institute (NHLBI) R01 HL152735 and R01 HL137995. The COPDGene project described was supported by Award Number U01 HL089897 and Award Number U01 HL089856 from NHLBI. The content is solely the responsibility of the authors and does not necessarily represent the official views of the NHLBI or the NIH.

## Author Disclaimer

The content is solely the responsibility of the authors and does not necessarily represent the official views of the NHLBI or the NIH.

## Conflict of Interest

The authors declare that the research was conducted in the absence of any commercial or financial relationships that could be construed as a potential conflict of interest.

## Publisher's Note

All claims expressed in this article are solely those of the authors and do not necessarily represent those of their affiliated organizations, or those of the publisher, the editors and the reviewers. Any product that may be evaluated in this article, or claim that may be made by its manufacturer, is not guaranteed or endorsed by the publisher.

## Figures and Tables

**Appendix A:** Effect of different SmCCNet weights on Correlated PageRank methods.

**SmCCNet Weights**	**α**	**ϵ**	**Seed**	**Subgraph size**	**Φ**	**ρ(p_value)**	**Φ + ρ**
**Sequential Correlated PageRank (1-10-10)**							
**1,10,10 (613 nodes)**	0.03	0.0001	2,137	341	0.502	–0.31 (6.18e-23)	
	0.08	0.0001	303	321	0.501	–0.31 (2.68e-23)	
	0.01	0.001	905	307	0.501	–0.29 (8.79e-21)	
**1,5,5 (794 nodes)**	0.1	0.0001	2,137	357	0.209	0.16 (8.29e-07)	
	0.01	0.1	303	362	0.194	0.16 (7.87e-07)	
	0.01	0.1	905	359	0.17	0.16 (9.09e-07)	
**Simultaneous Correlated PageRank (1-10-10)**							
**1,10,10 (613 nodes)**	0.04	0.0001	2,137	328	0.528	–0.31 (7.43e-23)	0.226
	0.01	0.001	303	385	0.552	0.32 (2.18e-24)	0.233
	0.1	0.000001	905	208	0.634	–0.34 (2.5e-28)	0.243
**1,5,5 (794 nodes)**	0.01	0.1	2,137	362	0.209	0.16 (9.02e-07)	0.086
	0.01	0.1	303	361	0.194	0.16 (8.08e-07)	0.089
	0.01	0.1	905	359	0.17	0.16 (9.09e-07)	0.094
